# Improving assent in health research: a rapid systematic review

**DOI:** 10.1186/s12874-020-01000-3

**Published:** 2020-05-13

**Authors:** Dominik Soll, Maria Magdalena Guraiib, Nigel Campbell Rollins, Andreas Alois Reis

**Affiliations:** 1 Department of Endocrinology and Metabolism, Charité Universitätsmedizin Berlin, Berlin, Germany; 2 Global Health Ethics Team, World Health Organization, Geneva, Switzerland; 3 Maternal, Newborn, Child and Adolescent Health, World Health Organization, Geneva, Switzerland

**Keywords:** Informed consent, informed assent, informed consent forms, minors, adolescents, children, comprehension, understanding, rapid review

## Abstract

**Background:**

Enrolment in a research study requires the participant’s informed consent. In the case of minors, informed consent of the respective legal guardian is obtained in conjunction with informed assent of the underage p

articipant. Since comprehension of the information provided may be limited, effective interventions to improve understanding should be identified. Thus, it is the objective of this study to review quantitative studies that tested interventions to improve the understanding of information provided during assent processes in health research. The studied population consisted of minors that participated or were willing to participate in research. The primary outcome was the level of comprehension after intervention.

**Methods:**

A systematic search was conducted in eleven databases including regional databases: PubMed, Web of Science, ERIC, PsycINFO, CINAHL, POPLINE, AIM, LILACS, WPRIM, IMSEAR, and IMEMR and included references from inception of the database until July 2018 except PubMed which spanned the period from May 2013 to July 2018. Search terms focused on Informed Consent/Assent, Minors, and Comprehension. To complement the search, reference lists of retrieved publications were additionally searched. We included all quantitative studies that were conducted in minors, tested an intervention, covered assent processes in health research, and assessed comprehension. One reviewer screened titles, abstracts, and full-texts to determine eligibility and collected data on study design, population, intervention, methods, outcome, and for critical appraisal. Interventions comprised enhanced paper forms, interspersed questions, multimedia format, and others.

**Results:**

Out of 7089 studies initially identified, 19 studies comprising 2805 participants and conducted in seven countries were included in the review. Fourteen studies (74 %) tested an intervention against control and ten (53 %) were randomized controlled trials. Heterogeneous methodology as well as incomplete outcome and statistical reporting impaired the reliability of the collected data. Positive effects were suggested for use of enhanced paper forms, interspersed questions, use of pie charts, and organizational factors.

**Conclusions:**

Improving assent in health research is an under-researched area with little reliable evidence. While some interventions are proposed to improve understanding in assent processes, further investigation is necessary to be able to give evidence-based recommendations.

**Trial registration:**

PROSPERO ID: 106808.

## Background

Out of 26 917 clinical trials that have been registered on ClinicalTrials.gov in 2019, more than 4 700 included individuals under the age of 18 years [1]. Similar to adults, adolescents and children have a right and interest to participate in health research to ultimately benefit from its outcome. Since minors may have difficulties balancing risks and benefits, they are considered a vulnerable population. Thus, special requirements have to be met to include them in research.

Based on the principle of respect for persons, involvement in a study requires the participant’s informed consent [2]. In the case of minors, obtaining informed consent of the respective legal guardian in conjunction with informed assent of the underage participant is required. Usually, the minor’s decision prevails. The Council for International Organizations of Medical Sciences (CIOMS) provides guidelines regarding the consent/assent process in matters of content and comprehension. Among other things, the information should cover the study’s aims, procedures, anticipated benefits and potential risks as well as the voluntariness of participation and the right to withdraw at any times [2]. It is also essential, however, to ensure that the potential participant sufficiently understands the information provided.

One report demonstrated that the comprehension of study details by minors often is unsatisfactory with about 50 % not remembering that their treatment was considered research a few months after enrolment in the studies [3]. Although there are publications that provide guidance in assent processes, the recommendations often lack evidence [4]. However, different and novel ways of communicating information to minors have lately been under investigation.

This review summarizes published data from quantitative studies examining assent processes to identify interventions that promote the highest level of understanding among minors in health research of the information provided. Thus, it is the aim of this paper to provide guidance to future researchers on how to develop more effective assent processes.

## Methods

This rapid systematic review was registered in PROSPERO 2018 (ID: 106808) and follows the Preferred Reporting Items for Systematic Reviews and Meta-Analyses (PRISMA) statement (Fig. S1).

### Eligibility criteria

We included studies that evaluated factors and interventions in assent processes for minors in health research in regard of their impact on understanding. However, studies were excluded if 1) the study did not include children or adolescents (< 18 years); 2) the tested assent form was not for health research; 3) no intervention was tested; 4) the produced data was purely qualitative or narrative or 5) comprehension was not tested. There were no restrictions in respect of language.

### Search strategy

The literature search was conducted in eleven databases: PubMed, Web of Science, ERIC, PsycINFO, CINAHL, POPLINE, AIM, LILACS, WPRIM, IMSEAR, and IMEMR.

The search strategies were designed with input from an expert librarian to cover publications about Informed Consent/Assent, Minors, and Comprehension (Table S1). The searches were conducted between 26/07/2018 and 03/08/2018. Since a prior review covered earlier publications, the search in PubMed was restricted to papers published since May 2013 [5]. For other databases, the search was not limited in time. Additional records were identified by perusing references of retrieved publications.

### Assessment of studies

One reviewer screened all titles and/or abstracts and assessed full-texts to determine eligibility. If no full-text was available, authors were contacted to gain access. In case of queries about the potential eligibility of a study, these were discussed with at least one of the other reviewers and a joint solution was found.

### Data collection, synthesis and critical appraisal

All included studies were read and data were extracted by one reviewer. For data collection and assessment of risk of bias, a form was created based on the Data Extraction and Assessment Template by The Cochrane Public Health Group [6]. The obtained information included data on study design, population, intervention, methods, outcome and critical appraisal, among other things. As preferred outcome, mean of overall correct answers in a post-intervention comprehension test and respective statistical appraisal were obtained; if possible, absolute values where transformed to relative values. Furthermore, studies were clustered according to the intervention tested: enhanced paper forms, interspersed questions, multimedia format, and others. Levels of evidence are as follows: (1) randomized controlled trial (RCT); (2) controlled trial without randomization or prospective comparative cohort trial; (3) case-control study or retrospective cohort study; (4) case series with or without intervention or cross-sectional study or study without control [7]. In case of queries about the data collection and appraisal process, these were discussed with at least one of the other reviewers and a joint solution was found.

## Results

**Fig. 1:**
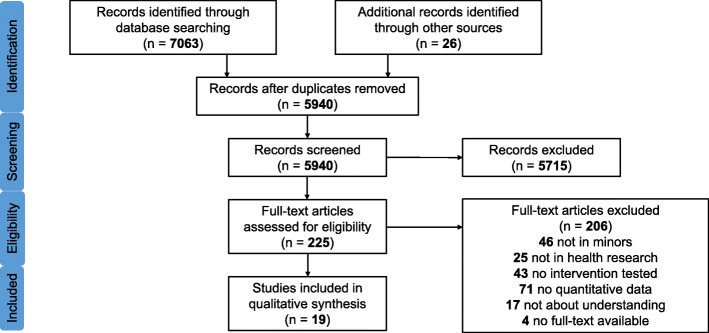
PRISMA flow chart for study selection

The primary search produced 7063 reports which were complemented by 26 reports obtained from other sources. After removal of duplicates and screening of titles and/or abstracts, 225 potentially relevant publications were identified and the full-texts were screened for eligibility. Application of exclusion criteria resulted in 19 studies with 2805 participants that were included in the analysis (Fig. 1).

### Study characteristics

**Fig. 2:**
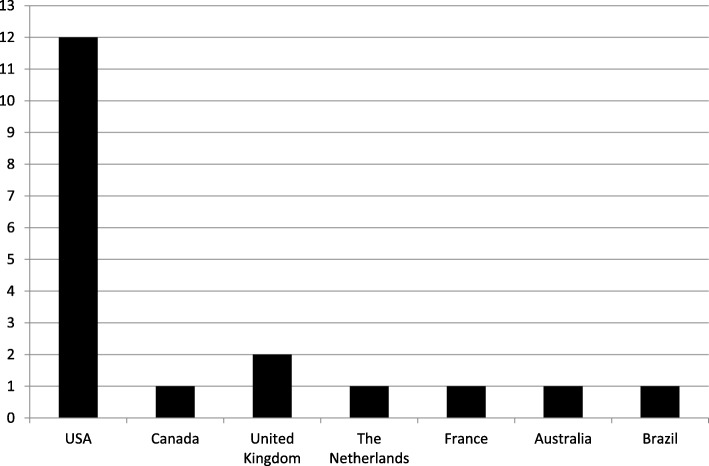
Regional distribution of included studies

The nineteen included studies were conducted in seven different countries (Fig. 2) and comprised ten (53 %) RCTs of which six (32 %) failed to clearly state the method employed for group allocation. Assessors were stated to be blinded only in two studies (11 %). Overall, fourteen studies (74 %) compared an intervention to a control and thirteen (68 %) used a standard assent process/form as control. A large variety of methods were used to assess understanding ranging from written questionnaires with multiple choice or open-ended questions in most studies to interviews and observations. Two studies (11 %) included less than five participants. Assent processes in real research settings were covered by seven studies (37 %) while the others (63 %) used hypothetical or simulated study protocols. In total, six studies (32 %) reported their outcomes incompletely. (Table 1)

**Table 1:** Evidence for comprehension in informed assent processes.

Authors	Study design	Intervention & Control	Outcome	Findings	Critical appraisal
Abramovitch et al. (1995) [8]	Non-RCT Participants: 177 healthy children (7-12 years) Description: 3 sub-studies on memory, hearing loss and personality Simulated scenario	Control: Standard descriptions of the sub-studies (n=131) Intervention: Standard descriptions + **probing questions** after descriptions of each study (n=46)	Measurement: Understanding of purpose, good things, and bad things Time point: after the description of all 3 studies	**With probing questions**, children understood **purpose and good things better than without interspersed questions**; no effect on understanding of bad things	Quality Rating: 2 Incomplete outcome reporting Non-comparable data provided Recruitment methods and selection unclear
Adcock et al. (2012) [9]	Crossover-RCT Participants: 217 school children (7-11 years) Description: studies on blood pressure and gastroesophageal reflux Simulated scenario	Control: Standard assent form with 2 pages in paragraph form (n=190) Intervention: **KidSent assent booklet** with 16 pages with sentences and pictures (n=195) First, participants read one of the documents; 3 days later, they read the other document	Measurement: Understanding of study purpose, risks, procedures, and right to withdraw Time point: immediately after reading the respective document	Mean of correct answers for **standard form 78.5%** and for **KidSent booklet 71.8%**; **significant difference** between both groups Other results: more children in KidSent booklet group had perfect scores (34,7%) than in standard form group (22,1%); most children stated that they understood the KidSent booklet better	Quality Rating: 1 2 different studies were covered by the control and intervention form; one might have been more difficult to understand Randomization methods partly unclear
Annett et al. (2017) [10]	RCT Participants: 64 healthy and ill adolescents (12-17 years) Description: clinical trial on asthma Real scenario	Control: Standard assent process with adolescent and parent together (n=34) Intervention: **Separate assent process** for adolescent and parent in different rooms (n=34)	Measurement: Understanding of asthma trial medicines, research process, rights and privileges, and risks and benefits Time point: immediately after assent process	In knowledge about risks and benefits, **minors over 15 years scored better when assent was separate** **Younger children showed no difference** Other results: Parents of older minors also showed better understanding when assent was separate; 15-17-year-olds scored better in asthma medicine than 12-14-year-olds	Quality Rating: 1 Incomplete outcome reporting Values for understanding between intervention and control not provided No description of randomization process
Barnett et al. (2005) [11]	RCT Participants: 374 school children (9-11 years, first language English) Description: in 7 schools; study how to convey concept of RCTs Simulated scenario	Control: Standard block text format (n=123) Interventions: (1) Question and answer (**Q&A**) format (n=126) and (2) **Story presentation** (n=124)	Measurement: Understanding of randomization, safety and effectiveness, voluntariness, and avenue of redress Time point: immediately after reading information	Mean of correct answers for **block text 70.4%**, for **Q&A 66.9%**, and for **story 64.2%** Other results: Significant difference for amount of participants that answered all questions per topic correct (story presentation scored best)	Quality Rating: 1 No statistics provided for comparison of mean scores No description of randomization process
Blake et al. (2015) [12]	RCT Participants: 120 adolescents (15-17 years, English-speaking, from youth serving agencies) Description: hypothetical HIV vaccine trial Simulated scenario	Control: Standard paper assent (n=31) Interventions: (1) Standard **paper assent with interspersed questions** (n=29) and (2) **Web-based assent with interspersed questions, videos, and clip arts** (n=60)	Measurement: Understanding of assent content Time point: immediately after assent process	Mean of correct answers for **paper assent 74.8%**, for **paper assent with questions 81.8%**, and for **web-based assent 78.1%**; **no significant differences** Other results: No significant difference for amount of participants that answered at least 80% correct (paper assent with questions best)	Quality Rating: 1 Less interaction with researcher in web-based assent Randomization methods stated
Chappuy et al. (2008) [13]	Retrospective interviews Participants: 29 ill children (HIV or cancer, 8,5-18 years) Description: participants recently recruited for other clinical trials Real scenario	Linking personal and disease factors and understanding of study processes	Measurement: Understanding of study purpose, protocol design and procedures, risks, direct and indirect benefits, right to withdraw, duration, alternatives, voluntariness Time point: after recruitment for respective clinical trial	Mean of correct answers when trial recruitment took place **more than 7 days after diagnosis 46%** and when it took place **earlier 20.8%; significant difference** between both groups Other results: adolescents older than 14 years scored better than younger ones	Quality Rating: 3 No controlled groups Inclusion of several different clinical trials may have influenced outcomes Small sample, potential bias by group imbalances
Coors et al. (2016) [14]	Non-RCT Participants: 76 healthy and ill adolescents (substance use disorder, 14-17 years, no intellectual deficiency) Description: biobanking and genomics study in several stages Real scenario	Control: Standard risk information Intervention: Standard risk information + **additional information on 7 previously identified salient risks**	Measurement: Understanding of risks Time point: immediately after assent process	**In patients**, the **additional information** on salient risks **improved scores significantly** **In healthy adolescents**, there was **no significant difference**	Quality Rating: 2 Incomplete outcome reporting No description of allocation of participants; numbers per group unclear
Friedman et al. (2016) [15]	RCT Participants: 568 healthy adolescents (14-17 years, male only, gay or bisexual) Description: survey on online behaviour of gay youth Real scenario	Control: Study information (n=186) Interventions: (1) Study information + **requirement to answer 2 questions correctly** (n=187) and (2) Study information + **requirement to answer 7 questions correctly** (n=195)	Measurement: Understanding of risks and voluntariness Time point: immediately after survey	Mean of correct answers for **information without questions 63%**, **with 2 questions 92.5%**, and **with 7 questions 93%**; **significant difference** between conditions with and without questions Other results: assent significantly rarer completed when questions interspersed	Quality Rating: 1 Questions to assess understanding at the end are the same as used in the intervention Online study with high number of dropouts Male participants only Randomization methods stated
Grootens-Wiegers et al. (2015) [5, 16]	Interventional study Participants: 101 school children (10-13 years) Description: comic about characteristics of research studies Simulated scenario	Intervention: **Comic strip** with information on medical research (n=101)	Measurement: Understanding of 8 research aspects Time point: after reading the comic strip	Mean of correct answers for **comic strip 83.0%**; best score for side effects, worst score for anonymity Other results: survey on user satisfaction	Quality Rating: 4 No control group Recruitment methods and selection unclear Dropouts not described
Lally et al. (2014) [17]	RCT Participants: 120 adolescents (16-19 years old, male/female who have sex with men) Description: consent and brochures on characteristics of an HIV vaccine trial Simulated scenario	Control: Standard informed consent (n=42) Interventions: (1) Informed consent **with 1-sided supplemental information** (presentation of pertinent facts) (n=39) and (2) Informed consent **with 2-sided supplemental information** (n=39) (presentation of common misconceptions and rebuttal with factual information)	Measurement: Understanding of randomization, interpretation of side effects, and unproven efficacy (part of consent and intervention brochures); understanding of non-brochure topics Time point: immediately after reading the information	Mean of correct answers for **consent alone 72.1%**, for **consent + 1-sided information 78.6%**, and for **consent + 2-sided information 80.2%**; **significant difference** between consent only and consent + 2-sided information for randomization and side effects Other results: no significant differences for topics not covered by the supplemental brochures	Quality Rating: 1 Some participants are older than 18 years Randomization methods stated 5-point Likert-type response scale potentially inappropriate for understanding items
Lee et al. (2013) [18]	Interventional study Participants: 123 adolescents (12-17 years) Description: study on Hepatitis B vaccination in youth Real scenario	Intervention: **Simplified assent form** with every day, non-medical language and supporting graphs in a Q&A format	Measurement: Understanding of procedure, randomization, future benefits, blinding, direct benefit, voluntariness Time point: immediately after reading the form	Mean of correct answers for the **simplified assent form 85.8%** Other results: 56.1% answered all questions correctly	Quality Rating: 4 No control group Dropouts not described
Mayne et al. (2017) [19]	Case series with intervention Participants: 2 children (3 years) Description: Story of a toymaker who makes science toys Simulated scenario	Intervention: **Interactive nonfiction narrative** (powerpoint with photos, clip arts, active buttons) on touch computer; concepts: dialogic reading, sustained shared thinking, cycle telling and retelling	Measurement: Understanding of research purpose and context, participatory rights, and consent Time points: 1 week before, 2 and 9 weeks after outreach	**Understanding** of the basic research concepts **improved or stayed high** after presentation of the interactive narrative	Quality Rating: 4 No control group Only 2 selected participants Incomplete outcome data due to erratic interest of participants
Miranda et al. (2017) [20]	Interventional study Participants: 42 hospitalized children (5-10 years, clinically stable) Description: study on vulnerability during illness and hospitalization Real scenario	Intervention: **Illustrated booklet** (text, images, illustrations for colouring)	Measurement: Understanding of research proposal Time point: during application of booklet	**All children understood the research proposal** Other results: Children wanted the booklet to be able to colour it	Quality Rating: 4 No control group Understanding was assessed only by “observations by researcher” Inconclusive outcome reporting
Murphy et al. (2007) [21]	RCT Participants: 187 healthy adolescents (15-19 years, male/female/ transgender, at risk for HIV, English-speaking) Description: study on HIV vaccination Simulated scenario	Control: HIVNET standard assent form (n=94) Intervention: Based on HIVNET version, but **reorganized, simplified text, implementation of illustrations** (n=93)	Measurement: Understanding of study details including procedures, benefits and risks Time point: immediately after assent process	Mean of correct answers for **standard version 71.7%** and for **illustrative version with simplified text 80.5%**; **significant difference** between both groups Other results: understanding of procedures and benefits was also significantly better in the intervention group; illustrative version with simplified text contained fewer words, fewer words per sentence, less passive voice, and had higher reading ease	Quality Rating: 1 No description of randomization process No indication of standard deviations Simplified text and illustrations are tested together Some participants are older than 18 years
O’Lonergan and Forster-Harwood (2011) [22]	RCT Participants: 170 children (11-14 years, no deficits in cognition, hearing, or vision, did not undergo procedures yet) together with parents Description: study involving common procedures in paediatrics (DXA and abdominal ultrasound) Simulated scenario	Control: Standard permission and assent process (n=87) Intervention: **Multimedia process** in Microsoft PowerPoint with same text like standard process but with hyperlinks to videos and voice-overs (n=83)	Measurement: Understanding of essential elements of the permission and assent process Time point: immediately after assent process	Mean of points for correct answers for **standard process 44%** and for **multimedia process 51.2%**; **significant difference** between both groups for total score, study procedures, and risks Other results: parents also scored significantly better with multimedia process; all participants overestimated their comprehension	Quality Rating: 1 No description of randomization process Incomplete outcome reporting (answers to some questions were not presented individually)
Tait et al. (2007) [23]	RCT Participants: 190 hospitalized children (7-17 years, no cognitive impairment, no emergent illness) Description: study on postoperative nausea and vomiting Simulated scenario	Control: Standard form including verbal explanation (n=95) Intervention: **Modified form with improved readability and processability** as well as use of bullets, bolding, increased font size, and pictures (also including verbal explanation) (n=95)	Measurement: Understanding of purpose of study, protocol, risks, direct and indirect benefits, alternatives, voluntariness, and freedom to withdraw Time point: immediately after assent process	Mean of points for correct answers for **standard form 60.4%** and for **modified form 68.5%**; **significant difference** between both groups Other results: differences between groups were higher in younger children; most children preferred modified form; all children overestimated their comprehension	Quality Rating: 1 No description of randomization process Large number declined participation; possibly selection bias of highly motivated children Assessors were blinded
Tait et al. (2012) [24]	Before and after study Participants: 4 children (8-14 years, from waiting room in hospital) Description: pilot study; trial on asthma Simulated scenario	Intervention: **3D modelled avatars present a dialogue** between a child and a doctor in an interactive program	Measurement: Pre- and post-intervention understanding of clinical trial, randomization, placebo, and blinded study; post-intervention understanding of elements of the study Time point: directly before and after using the program	Correct descriptions of the 4 terms from pre- to post-intervention: **25% to 50%, 0% to 0%, 0% to 50%, and 25% to 50%**; mean of points for correct answers about elements of the study 61.7%	Quality Rating: 4 No control group Only 4 participants
Tait et al. (2015) [25]	RCT Participants: 135 children (10-17 years, attendants of a paediatric clinic, no cognitive impairments, English-speaking) Description: study on general aspects of trials Simulated scenario	Control: Standard paper form (text only) (n=68) Intervention: **Interactive iPad program** in written and visual formats together with voice-over and interactive exercises with corrective feedback (content identical to standard form) (n=67)	Measurement: Understanding of clinical trial, participation, protocol, randomization, placebo, blinding, double-blinding, effectiveness, and informed consent Time point: immediately after reading the information	Mean of points for correct answers for **standard form 49.2%** and **for interactive program 64.7%**; **significant difference** between both groups Other results: most children preferred the interactive program over the standard form	Quality Rating: 1 Randomization methods stated Assessors were blinded
Ulph et al. (2009) [26]	Cross-sectional study Participants: 106 school children (7-11 years) Description: study on methods to convey probabilities in a cup game Simulated scenario	6 different formats were tested in all participants: (1) **verbal labels** (rare) (2) **percentages** (1%) (3) **pie charts** (4) **proportions as words** (1 in 100) (5) **proportions as notation** (1:100) (6) **mixed format**	Measurement: 3 trials to choose the highest probability shown for each format Time point: during the game	Mean of points for correct answers was **highest for pie charts** (90%), followed by verbal labels, percentages (79%), proportions as words (64%), proportions as notation (62.7%), and mixed format (43%)	Quality Rating: 4 Game may not represent complexity of medical research Only understanding of probability was tested Incomplete outcome reporting

### Interventions

Nine studies investigated the effects of using enhanced paper forms during the assent process. Enhanced forms included those with simplified text, illustrations, supplemental information, and narrative approaches. Six studies tested an enhanced assent form against the respective standard form as a control. Five of the six studies stated to use randomization for group allocation. Three of these studies found that the enhanced form resulted in significantly better understanding than the standard form, while one study found the opposite being the case [9, 17, 21, 23]. However, the one study describing the standard form to be more effective was the only one where the intervention and the standard form did not cover the same content, but were used for two different clinical trials [9]. A non-randomized study found the enhanced form to significantly improve understanding in adolescent patients suffering substance use disorder but to have no effect in the control group of healthy adolescents [14]. Another randomized study tested block text format against questions and answers (Q&A) as well as story format, but failed to provide statistical calculations for the means of correct answers. Instead, they only stated that the highest portion of participants that answered all questions correctly was in the story group [11]. Three additional studies found that using everyday language with graphs and Q&A format, an illustrated booklet, and a comic strip in the assent process generally led to good understanding of the research details, but the interventions were not tested against a standard or control format [16, 18, 20].

Three studies investigated the effects of using questions that assessed comprehension being interspersed during the assent process. All three studies compared the standard form with the same form plus probing questions during the process. Two studies used randomization for group allocation. Two studies found the understanding to be improved with interspersed questions during the assent process. However, one study considered the effect not to be significant with p = 0.055 [12], while the other study used the same questions for probing during the process and assessment of understanding after the process [15]. The third study described better understanding of study purpose and benefits with probing questions but failed to provide the respective data and statistics in the report [8].

Five studies investigated the effects of using multimedia formats during the assent process. Three studies were RCTs that tested a multimedia format against the standard form. One study found the multimedia approach to significantly increase comprehension levels compared to the standard form, while another study found no significant difference [12, 22]. Another RCT tested a multimedia format with interactive, interspersed exercises against the standard form and found the multimedia format to be significantly better in improving understanding in the adolescent participants [25]. Two additional studies tested a non-fiction narrative on a touch computer and a dialogue by avatars in a low number of participants without a control condition [19, 24]. Interestingly, one study included two three-year-old children who could already be successfully introduced to certain aspects of research [19].

Three more studies investigated the effects of other interventions during the assent process. One RCT showed that adolescents above the age of 15 years demonstrated higher levels of comprehension when the assent process for them and their parents was conducted separately, while younger children showed no differences [10]. In a cross-sectional study, several different methods to explain probabilities were tested against each other, demonstrating that illustrations as pie charts were easiest to understand for children, followed by verbal labels, percentages, proportions as words, and proportions as notation [26]. An observational study demonstrated in interviews that understanding of study details was better if the recruitment and assent process took place more than seven days after the respective diagnosis. However, participants from several different clinical trials were included in the study and not controlled for their allocation which might have influenced the observed effect [13].

## Discussion

This review includes nineteen studies of which twelve have not yet been covered by Grootens-Wiegers et al. in a former systematic review [5]. We made an effort to cover a broad spectrum by inclusion of many regional databases and the literature search was designed to particularly imbed literature from many different cultural backgrounds. Unfortunately, only one study from a low- and middle-income country met the inclusion criteria for this review, while all other included studies come from Organization for Economic Co-operation and Development (OECD) countries (Fig. 2).

In general, reliable data published on this subject was scarce. At study level, we identified several factors that limit the power of the presented results. These include the low number of RCTs and the failure to sufficiently report the group allocation processes. The nature of the assent process impeded blinding of group allocation, whereas the possible blinding of assessors was undertaken only in two trials. In five studies, interventions were not tested against control, at all. Additionally, many of the comprised studies featured incomplete outcome reporting. This included especially the failure to provide mean and standard deviation values as well as the lack of statistical analysis.

The format of a rapid systematic review was chosen to provide high-level evidence for health researchers that work with minors in a timely manner. This decision comes with limitations at review-level: only one author conducted the principal literature search and data collection. This may have resulted in incomplete retrieval of identified research. However, we made an additional effort to improve the quality of this review by inclusion of at least one other reviewer in any case of doubts during the process of literature screening, data collection, and critical appraisal. Unfortunately, the limitations at study-level impeded additional (meta-)analyses of the presented interventions.

To differentiate individual opinions and views from verifiable results, we decided to exclude qualitative studies from this review. Nevertheless, new ways to communicate information in research have been tested in qualitative studies, as well. Dockett et al. report how one child emphasized the importance of illustrations in information forms: ‘I just read the pictures.’ [27] Another report described the process of involving children in the development of information and assent forms. The children exclusively used active voice and named all function owners [28]. However, their effects on comprehension still needs to be assessed.

## Conclusions

This report on a rapid systematic review includes nineteen studies that investigated factors in research assent processes in order to improve comprehension in underage participants. Unfortunately, available data on this topic proved to be rare and several major limitations restrict the power of the findings, so that we did not attain our initial goal to be able to provide researchers explicit evidence-based recommendations.

Nevertheless, positive impact on children’s and adolescents’ comprehension of research information was suggested for enhanced paper forms (e.g. by simplified text or illustrations), for the use of interspersed questions, for assent processes that are conducted separately from parents for adolescents older than 15 years, for the use of pie charts to communicate probabilities, and if trial recruitment took place more than seven days after diagnosis. The positive effect of simplified language, illustrations, and narrative approaches in enhanced paper forms may not be surprising given that presentations using various visualizations are generally supposed to be easier to understand and to follow [29]. And just like repetition is a widely accepted tool to study and understand any topic, the shown benefit from interspersed questions that require participants to double-check their own comprehension is quite intuitive. Like the use of pie charts, whenever probabilities are meant to be conveyed, both techniques are easily included in any kind of information sheets.

Younger children might profit from elements that show no impact in older ones and vice versa. Adolescents might feel a greater responsibility for their decisions. In line with that, Hein et al. claimed that children from the age of twelve may already be capable of giving consent instead of assent [30]. Therefore, future research on this topic should consider testing different interventions in different age groups.

On the whole, the area of assent remains a largely under-researched issue. Further research and standardization of measures still remain necessary to be able to give stronger evidence-based recommendations.

## Supplementary information

**Additional file 1 Table S1**. Search strategies.

**Additional file 2.** PRISMA checklist.

## Data Availability

The datasets used and analyzed during the current study are available from the corresponding author on request.

## Abbreviations

Q&A: Questions and answers
RCT: Randomized controlled trial
OECD: Organization for Economic Co-operation and Development
CIOMS: Council for International Organizations of Medical Sciences
PRISMA: Preferred Reporting Items for Systematic Reviews and Meta-Analyses
