# Supplementary material for: Improving assent in health research: a rapid systematic review
Source: BMC Med Res Methodol. 2020 May 13;20:114. doi: 10.1186/s12874-020-01000-3 (PMC7222594; doi:10.1186/s12874-020-01000-3)
Supplement: Supplementary file 1 — Additional file 1 Table S1. Search strategies. [file 12874_2020_1000_MOESM1_ESM.docx]

Table S1. Search strategies

| Database  (Access date) | Search strategy |
| --- | --- |
| PubMed  (25/07/2018) | (((("Informed Consent"[Mesh] OR "Consent Forms"[Mesh] OR "Informed Consent"[TW] OR "Consent"[OT] OR “Consent document”[OT] OR "Consent"[TIAB] OR "Patient Information"[TIAB] OR "Patient Education as Topic"[Mesh] OR "Patient Participation"[Mesh] OR "Clinical Trials as Topic"[Mesh])  AND  ("Minors"[Mesh] OR "Child"[Mesh] OR "Adolescent"[Mesh] OR "Minor"[TW] OR "Child"[TW] OR "Adolescent"[TW] OR "Minors"[TW] OR "Children"[TW] OR "Adolescents“[TW]))  OR "Informed Consent By Minors"[Mesh]  OR "Assent"[TW] )  AND (understanding OR understand* OR perception* OR apprehension OR motivation))  AND ("2013/05/02"[PDAT] : "3000/12/31"[PDAT]) |
| Web of Science  (31/07/2018) | (consent OR assent)  AND (child OR children OR minor OR minors OR adolescent OR adolescent)  AND (unterstanding OR understand OR comprehension OR motivation OR apprehension OR perception) |
| ERIC  (03/08/2018) | (MH "Consent (Research)")  AND minor OR minors OR child OR children OR adolescent OR adolescents  AND understanding OR comprehension OR understand OR comprehend OR motivation OR perception OR apprehension  OR assent |
| PsycINFO  (03/08/2018) | (MH "Consent (Research)")  AND minor OR minors OR child OR children OR adolescent OR adolescents  AND understanding OR comprehension OR understand OR comprehend OR motivation OR perception OR apprehension  OR assent |
| CINAHL  (26/07/2018) | (MH "Consent (Research)")  AND minor OR minors OR child OR children OR adolescent OR adolescents  AND understanding OR comprehension OR understand OR comprehend OR motivation OR perception OR apprehension  OR assent |
| POPLINE  (26/07/2018) | (consent OR assent)  AND  (child OR children OR minor OR minors OR adolescent OR adolescents)  AND  (understand OR understanding OR comprehension OR perception OR motivation) |
| AIM  (26/07/2018) | Consent OR assent OR patient information *in Titles, Keywords for all material types* |
| LILACS  (26/07/2018) | (consent OR assent) AND (child OR children OR minor OR minors OR adolescent OR adolescents) AND (understand OR understanding OR comprehension OR perception OR motivation) |
| WPRIM  (31/07/2018)  *7 individual searches* | Assent  Consent AND adolescent  Consent AND adolescents  Consent AND minor  Consent AND minors  Consent AND child  Consent AND children |
| IMSEAR  (31/07/2018) | (consent OR assent) AND (understand OR understanding OR comprehension OR perception)  *Subject Contains “*Adolescent*”* |
| IMEMR  (31/07/2018)  *7 individual searches* | Assent  Consent AND adolescent  Consent AND adolescents  Consent AND minor  Consent AND minors  Consent AND child  Consent AND children |
